# Unraveling the Extracellular Metabolism of Immortalized Hippocampal Neurons Under Normal Growth Conditions

**DOI:** 10.3389/fchem.2021.621548

**Published:** 2021-04-16

**Authors:** Beatrice Campanella, Laura Colombaioni, Riccardo Nieri, Edoardo Benedetti, Massimo Onor, Emilia Bramanti

**Affiliations:** ^1^ National Research Council, Institute of Chemistry of Organometallic Compounds (CNR-ICCOM), Pisa, Italy; ^2^ National Research Council, Institute of Neuroscience (CNR-IN), Pisa, Italy; ^3^ Hematology Unit, Department of Oncology, University of Pisa, Pisa, Italy

**Keywords:** metabolomics, primary cultured hippocampal neurons, HPLC-DAD, GC-MS, reproducibility

## Abstract

Metabolomic profiling of cell lines has shown many potential applications and advantages compared to animal models and human subjects, and an accurate cellular metabolite analysis is critical to understanding both the intracellular and extracellular environments in cell culture. This study provides a fast protocol to investigate *in vitro* metabolites of immortalized hippocampal neurons HN9.10e with minimal perturbation of the cell system using a targeted approach. HN9.10e neurons represent a reliable model of one of the most vulnerable regions of the central nervous system. Here, the assessment of their extracellular metabolic profile was performed by studying the cell culture medium before and after cell growth under standard conditions. The targeted analysis was performed by a direct, easy, high-throughput reversed-phase liquid chromatography with diode array detector (RP-HPLC-DAD) method and by headspace solid-phase microextraction–gas chromatography–mass spectrometry (HS-SPME-GC-MS) for the study of volatile organic compounds (VOCs). The analysis of six different batches of cells has allowed to investigate the metabolic reproducibility of neuronal cells and to describe the metabolic “starting” conditions that are mandatory for a well-grounded interpretation of the results of any following cellular treatment. An accurate study of the metabolic profile of the HN9.10e cell line has never been performed before, and it could represent a quality parameter before any other targeting assay or further exploration.

## Introduction

Biomedical research, from drug development to biotechnological production, involves the employment of cell line cultures (León et al., 2013). Cultured cell models in metabolomics are widespread employed in many areas of medical research as a valuable alternative to the use of animals in toxicology testing with benefits in term of a greater control of external variables and no ethical problems. The study of metabolites in cell cultures can provide information on the reactivity of cells to external *stimuli* (Zhang et al., 2013). Thus, standard procedures for cultured mammalian cell metabolomics are researched worldwide (Hayton et al., 2017; Ivanisevic and Want, 2019; Villaret-Cazadamont et al., 2020), and many authors claim in their concluding comments that standardized procedures are still needed (Hayton et al., 2017; Hirsch and Schildknecht, 2019).

Mammalian cells suffer from high variability, which has to be controlled and investigated in order to get reliable and reproducible results once the cell cultures undergo specific treatments (Wright Muelas et al., 2018). The metabolic state of the cells depends on nutrients’ concentrations, oxygenation, pH, and cell density (Cook et al., 2014; Wright Muelas et al., 2018), and cell metabolism is critical in cell growth and survival. The metabolic performance of the cell lines can be a prime cause of irreproducibility of experiment outcome.

Given the great potential in the application of mammalian cell culture metabolomics in toxicological studies, the investigation of their normal metabolic profile and its consistency in untreated cell cultures is mandatory for a well-grounded interpretation of the cellular treatment effects. The characterization of basal metabolites could also be helpful to define, for toxicological experiments, the “starting time point,” which is currently based on the observation of the confluence value after a standardized procedure.

Thus, the development of effective, robust, standard procedures to generate cultured cell samples for metabolomic analysis and the need of low-cost, straightforward methods to check the goodness of cell samples for deeper metabolic investigations are of interest.

Cell culture metabolomic studies are focused on both intracellular metabolites from isolated cells and extracellular metabolites released by cells in the cell culture medium (CCM). Aurich et al. (2015) applied the extracellular metabolic data to the prediction of intracellular metabolic states in lymphoblastic leukemia cell lines. Extracellular metabolite analysis can give a picture of the metabolic state inside the cell and suggest potential metabolic pathways. Despite the limitation of having a picture of only extracellular metabolites, resulting from the interchange (uptake of substrates/excretion) of metabolic products, between cells and the CCM, the analysis of CCM has several advantages. First, the analysis of CCM of living cells guarantees a minimal sample handling of cells to avoid artifacts. Second, CCM reflects the metabolic activity of cells in response to experimental perturbations without cell disruption, thus allowing the monitoring of the metabolic changes over time in the same cell culture. Third, in several conditions (e.g., adherent cell lines), the CCM can be rapidly collected, diluted, filtered, and analyzed, avoiding long and manifold extraction procedures (e.g., cell lysis).

Recently, we published a study on the metabolic implications on the neuronal metabolism of the HN9.10e cell line following a short and transient exposure to low thallium chloride doses, based on the chromatographic analysis of CCM and on morphological and functional tests (Colombaioni et al., 2017b).

This neuronal cell line presents structural and functional features analogous to primary hippocampal neurons and represents a reliable *in vitro* model of one of the most vulnerable regions of the central nervous system (Lee et al., 1990). The HN9.10e cell line is well characterized, and it allows us to consistently evaluate minute functional alterations (Colombaioni et al., 2002; Voccoli et al., 2007; Voccoli and Colombaioni, 2009; Colombaioni et al., 2017a; Bramanti et al., 2019). A single 48 h exposure to 1, 10, and 100 μg/L Tl^+^ was found to have significant effects on neuronal growth rate and morphology (Colombaioni et al., 2017b; Bramanti et al., 2019) as well as on lactate and ethanol concentration in CCM. The increased production of these two metabolites was found to be associated with signs of cellular deregulation such as neurite shortening, loss of substrate adhesion, increase of cytoplasmic calcium, and dose-dependent alteration of mitochondrial ROS (mtROS) level and of transmembrane mitochondrial potential (ΔΨm) (Bramanti et al., 2019).

In this work, we propose the study of the extracellular metabolites of living immortalized hippocampal neuron (HN9.10e cell line) cell cultures in six replicated independent experiments by analyzing the cell-free culture medium (CFCM) and the cell culture medium (CCM) by a direct, easy, high-throughput reversed-phase liquid chromatography with diode array detector (RP-HPLC-DAD) method. Headspace solid-phase microextraction–gas chromatography–mass spectrometry (HS-SPME-GC-MS) of volatile organic compounds (VOCs) was also applied (Campanella et al., 2020) to confirm several possible metabolic pathways (Baranska et al., 2013, Baranska et al., 2015; Filipiak et al., 2016). No data are reported on the metabolic analysis of neurons through CCM.

Many of the mentioned works present in the literature and references therein reported on cell cultures are related to the study of metabolic effects due to various treatments of cell lines (Zhang et al., 2013; Aurich et al., 2015; Hayton et al., 2017; Ivanisevic and Want, 2019). Few articles investigated the metabolic reproducibility when cells are grown under normal conditions (León et al., 2013; Wright Muelas et al., 2018).

This study also aims to face with the metabolic reproducibility of neuronal cells and to describe the metabolic “starting” conditions for any experiment based on the cell culture model. The optimal condition to start an experiment is currently based on the observation of morphological features and the confluence value after a standardized procedure. The chromatographic investigation of cell normal metabolic profile could be a second-level quantitative check, and its consistency in untreated cell cultures is mandatory for a well-grounded interpretation of the results of any following cellular treatment.

This approach, based on the relative simplicity of measuring the variations of chemical composition in CCM, complies with the goal of detecting as many metabolites as possible and of minimizing sample handling procedures and the number of analyses.

## Materials and Methods

### Chemicals and Procedures

Sulfuric acid for HPLC analysis was employed (30,743 Honeywell Fluka 95–97%). Methanol for RP-HPLC was purchased from Merck (34,860, ≥99.9%).

Standard solutions for HPLC (TraceCERT^®^, 1,000 mg/L in water) were purchased from Sigma-Aldrich (Milan, Italy). Analyte stock solutions were prepared by dissolving a weighed amount of the pure compound in deionized water and stored at 4°C up to 1 month. Ethanol, ^13^C-labeled ethanol, acetoin, acetone, butanal, 2-methylbutanal, 3-methylbutanal, butanedione, butanol, 2-methyl-butanol, 3-methyl-butanol, butanone, hexanal, methanol, methylacetate, 2-pentanol, propanal, 2-methylpropanal, propanol, 2-methyl-propanol, and sec-butanol were purchased from Sigma-Aldrich (Italy). All chemicals, having purity higher than 99%, were used without any further purification.

Solid-phase microextraction fiber based on 85 um carboxen/polydimethylsiloxane (CAR/PDMS) was employed for the preconcentration of volatile compounds in the HS.

Helium 5.6 IP was purchased from SOL Group Spa (Italy) and was further purified with a super clean filter purchased from Agilent Technologies (United States) to remove water, oxygen, and hydrocarbon contaminants.

Preparation/dilution of samples and solutions was performed gravimetrically using ultrapure water (Milli-Q; 18.2 MΩ cm^−1^ at 25°C, Millipore, Bedford, MA, United States).

### Cell Culture and Automated Imaging of HN9.10e Neuroblasts

The HN9.10e cell line was originally developed by Lee and Wainer by immortalization of murine hippocampal neuroblasts through the somatic cell fusion with N18TG2 neuroblastoma cells (Lee et al., 1990). The HN9.10e cell line aliquot after thawing was grown in HEPES buffered DMEM-F12 (1:1) medium, supplemented with 25 mM glucose, 4 mmol/L glutamine, 50 UI/ml penicillin, and 50 mg/ml streptomycin (named CCM), at 37°C in a humidified atmosphere containing 5% CO_2_ and cultured for 7 days at 37°C up to 0.4 confluence. After this, adherent cells were resuspended, and 1 ml (cell density around 330.00 cell/ml) of the cells was seeded at 20,000 cell/cm^2^ in culture flasks containing 5 ml of fresh CCM (*N* = 6 replicates) and left in culture for 4 days before removing the CCM to allow substrate adhesion and growth to an optimal 40% confluence. After this, the CCM was removed from each flask and analyzed by RP-HPLC-DAD and HS-GC-MS. The cell-free culture medium (CFCM) was also analyzed (*N* = 6 replicates).

To analyze the cell morphology and growth rate, avoiding the metabolic alterations or the viability loss potentially induced by cell dyes, an inverted microscope (Axiovert 35, Carl Zeiss, Oberkochen, Germany) equipped with Nomarski interference contrast optics and 40x or 63x objective lens was used. This setup allowed us to monitor the growth and death events in unstained living cultures, without the need for any dye or fluorescent probe.

The degree of cell confluence was evaluated by the automated measure of the ratio (surface occupied by cells/cell-free surface) obtained with a dedicated routine of MATLAB scientific software (The MathWorks, Massachusetts, MA, United States) adapted to the cell shape and contrast level of HN9.10e cultures. The measurements have been performed in five independent, nonoverlapping fields (400 × 400 μm) for each flask. At the time of the analysis, a confluence value of 0.4 was selected and each field contained, on average, 150 cells. The results were expressed as mean ± SD, and statistical significance of the differences was assessed by two-way ANOVA for a normal distribution of data. A *p* value of < 0.01 was considered as significant.

### Targeted Metabolomics, Data Processing, and Statistical Analysis

The cell culture medium was analyzed by RP-HPLC-DAD and HS-SPME-GC-MS (Campanella et al., 2019, Campanella et al., 2020). Details regarding both methods and acquisition parameters are listed in Supplementary Tables S1, S2. For HS-GC-MS analysis, 1 ml of the sample was removed from the flasks and collected in 10 ml headspace vials. The vials were sealed with holed screw caps equipped with Teflon/silicone septa for use with the CombiPAL and kept at −20°C until the analysis. The transfer of such volumes was accomplished using adjustable pipettes and, for better precision, all aliquots were weighted. Analytes were identified and quantified on the basis of their retention time (+0.1% variability) and the ratio between the quantifier and qualifier ion (+10% variability). The chromatographic peaks for targeted metabolites and the deuterated internal standard were detected (Supplementary Table S3), integrated by the GC/MSD ChemStation software (version E.02.02; Agilent Technologies, United States), and then checked manually. The peak–area ratio to the internal standard was calculated, and the concentration was obtained by building a calibration curve with the corresponding analytical standard. A representative GC-MS chromatogram of a CCM sample is reported in Supplementary Figure S1.

For RP-HPLC-DAD analysis, CCM samples were diluted 5 times in 5 mM sulfuric acid, filtered using a 0.20-μm RC Mini-UniPrep (Agilent Technologies, Italy), and injected in the HPLC system (V_inj_ = 5 μL). The identification of metabolites was based on the comparison of the retention time and UV spectra of standard compounds. The 220-nm detection was selected to control the interference of high absorbing compounds, the signals were manually integrated, and the concentration was obtained by building a calibration curve with the corresponding analytical standard (Supplementary Table S4).

All data were entered in a table, and the missing values were replaced with random numbers between 0 and the limit of detection of the specific analyte. Univariate statistical analysis was performed using R v2.13.0 (R Development Core Team, 2010; cran.r-project.org). Significant differences between CFCM and CCM were assessed with Student’s t test (unpaired, two-tailed) after Benjamini–Hochberg correction for multiple comparisons. *p* values  < 0.05 were considered significant.

## Results

**FIGURE 1:**
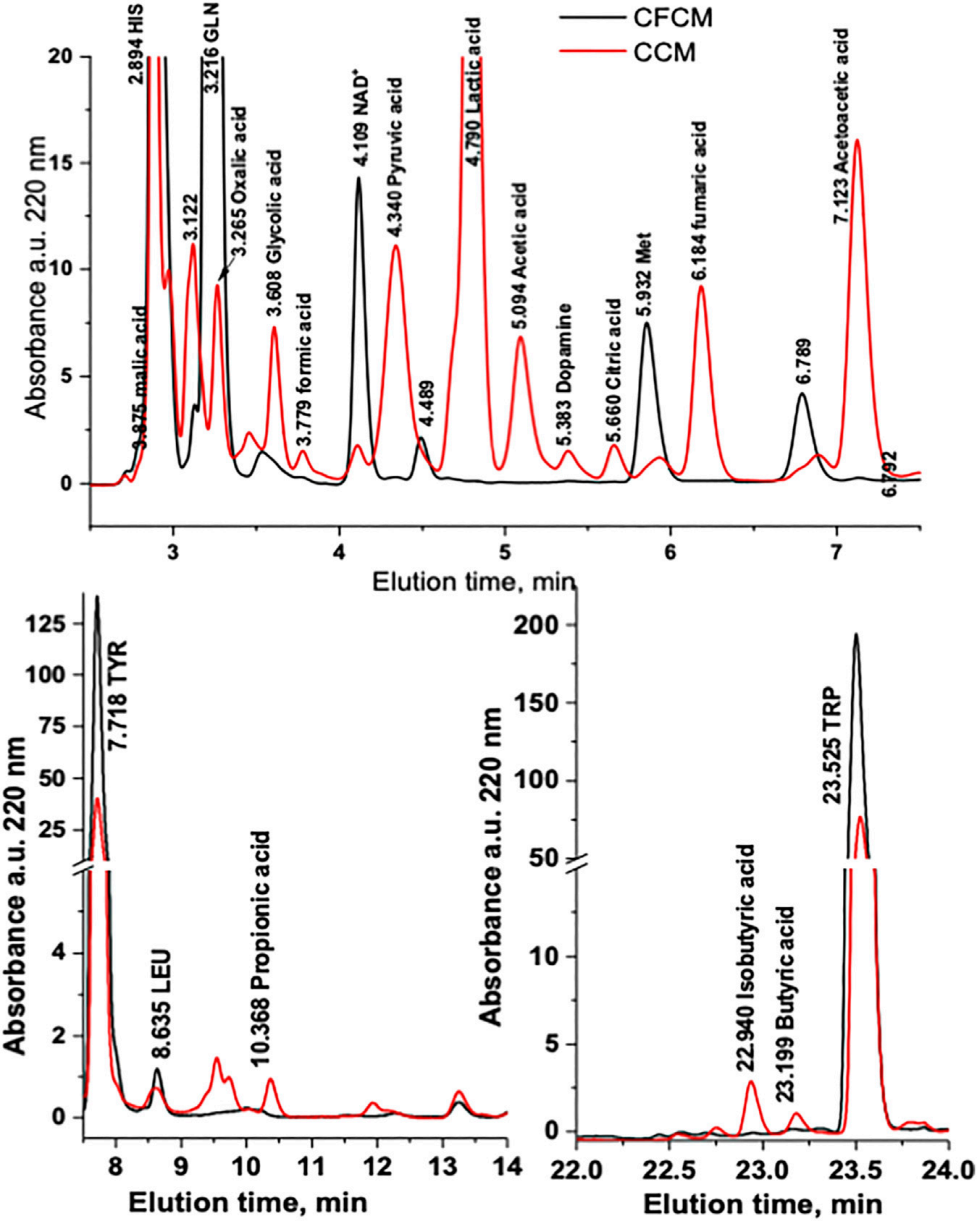
Representative absorbance chromatogram at 220 nm of the CFCM and CCM after 4 days of HN9.10e cell line culturing (V_inj_ = 5 μL). The chromatogram of the blank has been subtracted. 1 = L-histidine; 2 = L-threonine/L-glutamic acid; 3 = L-glutamine; 4 = oxalic acid; 5 = L-cysteine; 6 = glycolic acid; 7 = formic acid; 8 = NAD^+^; 9 = pyruvate; 10 = lactic acid; 11 = acetic acid; 12 = dopamine; 13 = citrate; 14 = L-methionine; 15 = fumaric acid; 16 = succinic acid; 17 = acetoacetic acid; 18 = L-tyrosine; 19 = L-leucine; 20 = propionic acid; 21 = L-phenylalanine; 22 = isobutyric acid; 23 = butyric acid; 24 = L-tryptophane.

In this work, the normal metabolism of the HN9.10e neuronal cell line under standard growing conditions was assessed through the analysis of CFCM and CCM by RP-HPLC-DAD and HS-SPME-GC-MS. Figure 1 shows the comparison of representative absorbance chromatograms at 220 nm of CFCM and of CCM after 4 days of cell culturing. The considerable reproducibility of the analysis obtained on six independent cultures is shown in Supplementary Figure S2.

**FIGURE 2:**
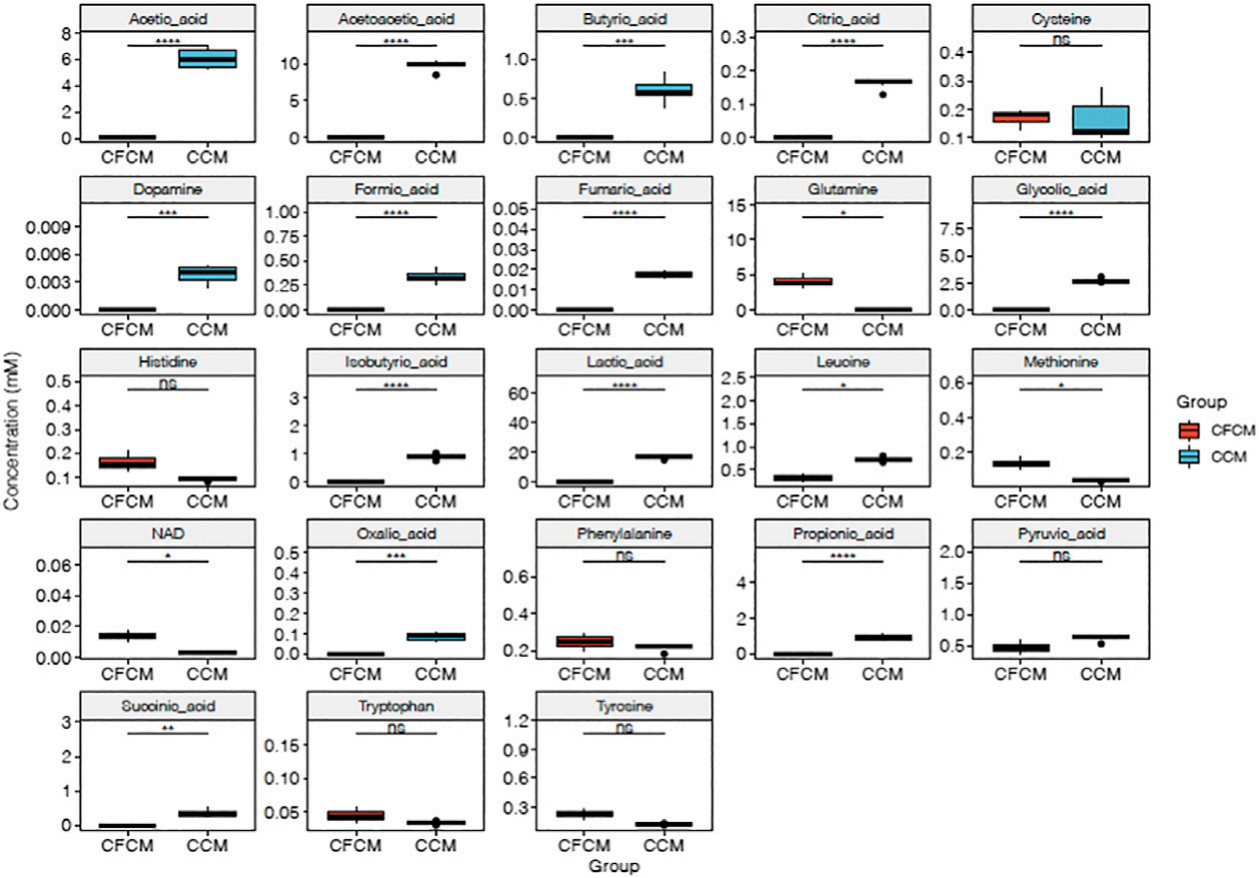
Boxplots of RP-HPLC-DAD autoscaled and mean-centered data, showing the differences between metabolites determined by RP-HPLC-DAD in the CFCM and CCM (ns *p* > 0.05; *p ≤ 0.05; **p ≤ 0.01; ***p ≤ 0.001; ****p ≤ 0.0001).

Figure 2 shows the boxplots of absolute quantification of selected metabolites (autoscaled and mean-centered) determined by RP-HPLC-DAD in the CCM before and after 4 days of HN9.10e cell line culturing. The differences between the two groups are evident (Figure 2; Supplementary Table S6). In the cell-free CCM, 25 metabolites can be potentially determined. However, because of the close retention time, L-Thr and L-Glu were not determined in the CCM after cell growth. An unpaired Student’s t test was performed to confirm the significant differences in the mean values of each metabolite.

**FIGURE 3:**
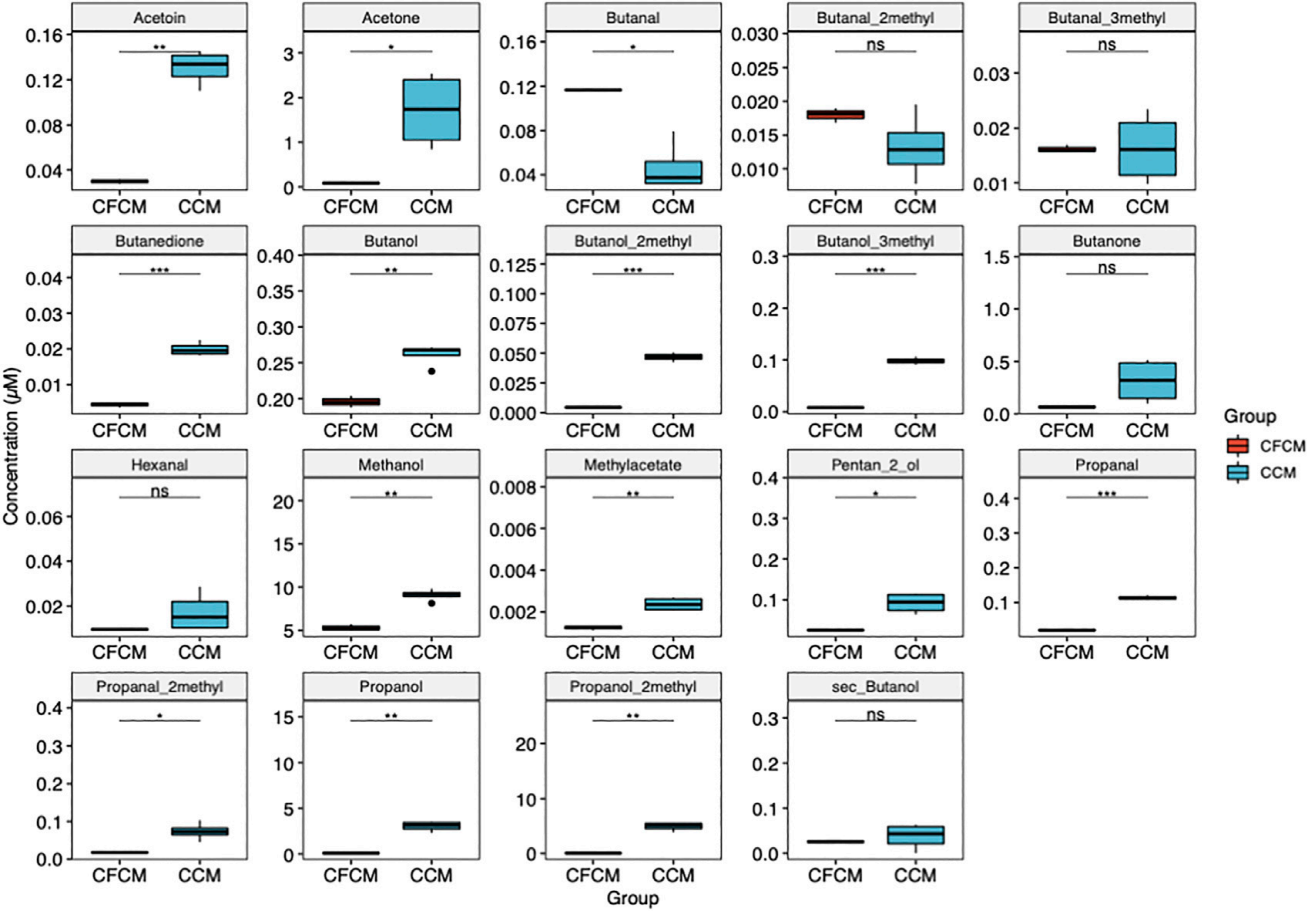
Boxplots showing the differences between volatile metabolites determined by HS-GC-MS in the CCM before and after 4 days of HN9.10e cell line culturing (ns *p* > 0.05; *p ≤ 0.05; **p ≤ 0.01; ***p ≤ 0.001; ****p ≤ 0.0001).

All volatile metabolites determined by HS-SPME-GC-MS increased significantly in the CCM after cell culturing (Figure 3), except for butanal which decreased in the CCM likely due to its oxidation to butanol. Ethanol and isopropanol were not quantified because of environmental contamination due to sanitizing agents employed for COVID-19 emergency.

All *p* values (before and after adjustment for multiple comparison) were significantly lower than 0.05 (Figures 2, 3), except for L-cysteine, L-histidine, L-tryptophane, and L-tyrosine. L-histidine, L-tryptophane, and L-tyrosine were quantified in the CFCM giving recoveries of 110, 102, and 106%, respectively, with respect to the concentration declared. In the CCM after 4 days of HN9.10e cell line culturing, their concentration showed a decreasing trend.

## Discussion

After 4 days of cell culturing, two amino acids of CFCM (L-methionine and L-glutamine) and niacinamide determined as NAD^+^ were significantly consumed (by 100% L-methionine and 100% L-glutamine). The total consumption of L-glutamine and L-methionine is a fingerprint of immortalized replicating cells. The decreasing trend for L-histidine, L-tyrosine, L-phenylalanine, and L-tryptophane concentrations is compatible with the employment of these amino acids as a precursor of L-glutamic acid, which is the principal excitatory neurotransmitter of the mammalian brain (Polis and Samson, 2020), dopamine, a monoamine neurotransmitter involved in the stability of hippocampal-dependent memory (McNamara et al., 2014), and several neuroactive compounds such as 5-hydroxytryptamine, kynurenines, and melatonin, respectively (Ruddick et al., 2006; Höglund et al., 2019). Dopamine concentration increases in the CCM after neuron culturing reaches indeed 3.8 μM.

NAD^+^ is an important energy substrate and cofactor involved in multiple metabolic reactions, and its level is a critical determinant of neuronal survival (Liu et al., 2008; Aman et al., 2018; Hou et al., 2018; Yoshino et al., 2018). Here, the observed NAD^+^ decrease may be related to its use in the tricarboxylic acid cycle (TCA).

In addition to the increase in dopamine and L-leucine, 12 metabolites significantly increased: oxalic acid, glycolic acid, formic acid, lactic acid, acetic acid, citric acid, fumaric acid, succinic acid, acetoacetic acid, propionic acid, isobutyric acid, and butyric acid. These results were consistent along six independent replicates (Supplementary Figure S2; Supplementary Table S6).

The increase from 325.1 ± 90.6 to 711.4 ± 45.7 μM was also observed for the neutral, branched L-leucine, which is known to be involved, as well, in the neuronal signaling process (Heeley et al., 2018) and in the regulation of glutamate levels, the major excitatory neurotransmitter in the central nervous system (Hutson et al., 2001).

Four metabolites that increased their concentration level after 4 days of HN9.10e cell line culturing (lactic, fumaric, succinic, and citric acids) are related to glycolysis and TCA cycle intermediates, indicating a strong metabolic activity of the growing cells. The remaining metabolites can be classified as short-chain fatty acids (SCFAs, i.e., formic, acetic, propionic, butyric, and isobutyric acids), ketone bodies (acetoacetic acid), C2 hydroxy acid (glycolic acid), and C2 dicarboxylic acid (oxalic acid).

Lactic acid, the main metabolite of CCM after cell culturing (17.1 mM), can be derived from glucose and L-glutamine metabolism (Divakaruni et al., 2017). TCA cycle intermediates, citric, fumaric, and succinic acids, were found to increase in the extracellular medium reaching concentration levels of 161.6 ± 16.6, 17.5 ± 1.5, and 350.4 ± 111.4 μM, respectively. A reasonable hypothesis is the release of these metabolites in the extracellular medium by means of the monocarboxylate transporters (MCTs) (Pierre and Pellerin, 2009).

Glycolic or hydroxyacetic acid (2.7 ± 0.171 mM in the CCM after the neuron culturing) is the smallest alpha-hydroxy acid, and it is well known to be derived from gastrointestinal yeast overgrowth, bacterial species, or dietary sources containing glycerol (Wishart et al., 2018). However, glycolic acid could arise in cells from L-glycine (249.8 mM in CFCM) and glyoxylate metabolism through glyoxalases (Meléndez-Hevia et al., 2009; Toyoda et al., 2014; Wishart et al., 2018). Glyoxalases are enzymes that can transform 2-oxoaldehydes, glyoxal, and methylglyoxal, into the corresponding 2-hydroxy acids, glycolate, and D-lactate, respectively (Toyoda et al., 2014). In this work, the high concentration of lactate (17.1 mM), which in our operating conditions cannot be speciated in L- or D-lactate, supports this hypothesis. In addition to glycolate, intermediate glyoxylate can be metabolized to formate and oxalate (337.3 ± 60 and 85.3 ± 20 μM, respectively, in the CCM after neuron culturing) and CO_2_ (Wishart et al., 2018). In cytosol, glyoxylate reductase–hydroxypyruvate reductase catalyzes the conversion from glyoxylate to glycolate through the NADPH-dependent reaction (Cochat and Rumsby, 2013) and a small amount of glyoxylate is also converted into oxalate by cytoplasmic lactate dehydrogenase (Wishart et al., 2018). In peroxisome, glycolic acid can be metabolized to oxalic acid via glycolate oxidase (Wishart et al., 2018). Under certain circumstances, glyoxylate can be a nephrotoxin and a metabotoxin and as an aldehyde. Glyoxylate is also highly reactive and will modify proteins to form advanced glycation products (AGEs) (Cochat and Rumsby, 2013).

The detection of SCFAs at millimolar and submillimolar concentration levels in the CCM after 4 days of HN9.10e cell line culturing is interesting and intriguing (formic acid, 0.337 ± 0.06; acetic acid, 6.1 ± 0.82 mM; propionic acid, 0.936 ± 0.127; isobutyric acid, 0.909 ± 0.092; and butyric acid, 0.600 ± 0.158 mM). SCFAs can be derived from the mitochondrial metabolism of L-valine, L-leucine, and L-isoleucine amino acids (de Koning et al., 2007; Polis and Samson, 2020). Their intermediate metabolites can serve as substrates in various vital biological processes, such as cholesterol, fatty acid synthesis, and Kreb’s cycle (Freeman and Learning, 2017).

It is well known that SCFAs are produced by several fermentation processes of obligate anaerobic bacteria, and it is universally accepted that in humans, the only significant sources of SCFAs are the microbiota and the ingestion of dairy products (Dalile et al., 2019). It is known, as well, that mammalian cells do not produce SCFAs.

Neurons are very sensitive to butyric acid and its derivatives (e.g., gamma-amino butyric acid, GABA, or drugs such as valproic acid and 4-phenylbutyric acid drugs) (Cayo et al., 2009; Kim et al., 2013; Cueno et al., 2015; Sharma et al., 2016; Sadler et al., 2020). Shah et al. reported that butyric acid can regulate tyrosine hydroxylase (TH) mRNA levels in a PC12 cell model (Shah et al., 2006), and Nankova et al. reported that SCFAs such as propionic acid and butyric acid produced by gastrointestinal bacteria are involved in the development of neuronal disorders including autism spectrum disorders influencing brain monoaminergic pathways (gut–brain axis) (Nankova et al., 2014). It is also known that clinical effects of propionic acidemia are largely neurological (Nguyen et al., 2007) and inflammation related (Abdelli et al., 2019).

All these results refer to the effects of exogenous SCFAs. However, recently we found that endogenous butyric acid increases up to 2.7 mM in HN9.10e immortalized neurons treated with 50 and 75 mM glucose (Colombaioni et al.2017a). Considering the mitochondrion’s origin, that is, bacteria (Gray et al., 1999), it can be hypothesized that these small metabolites are correlated to the response of the cell to a physiological (cell growth) or an external stress (e.g., hyperglycemia or treatment toxic agents), which is involved in an alteration of energetic metabolism (Colombaioni et al., 2017a; Bramanti et al., 2019), and thus have an active role in neuron signaling.

Acetoacetate, one of the main ketone bodies produced during ketogenesis, was significantly increased in the CCM, up to 9.8 ± 0.66 mM. This high concentration found and the high concentration of acetic acid suggest an excess of acetyl-CoA, and it should protect against oxidative glutamate toxicity (Massieu et al., 2003; Noh et al., 2006).

**FIGURE 4:**
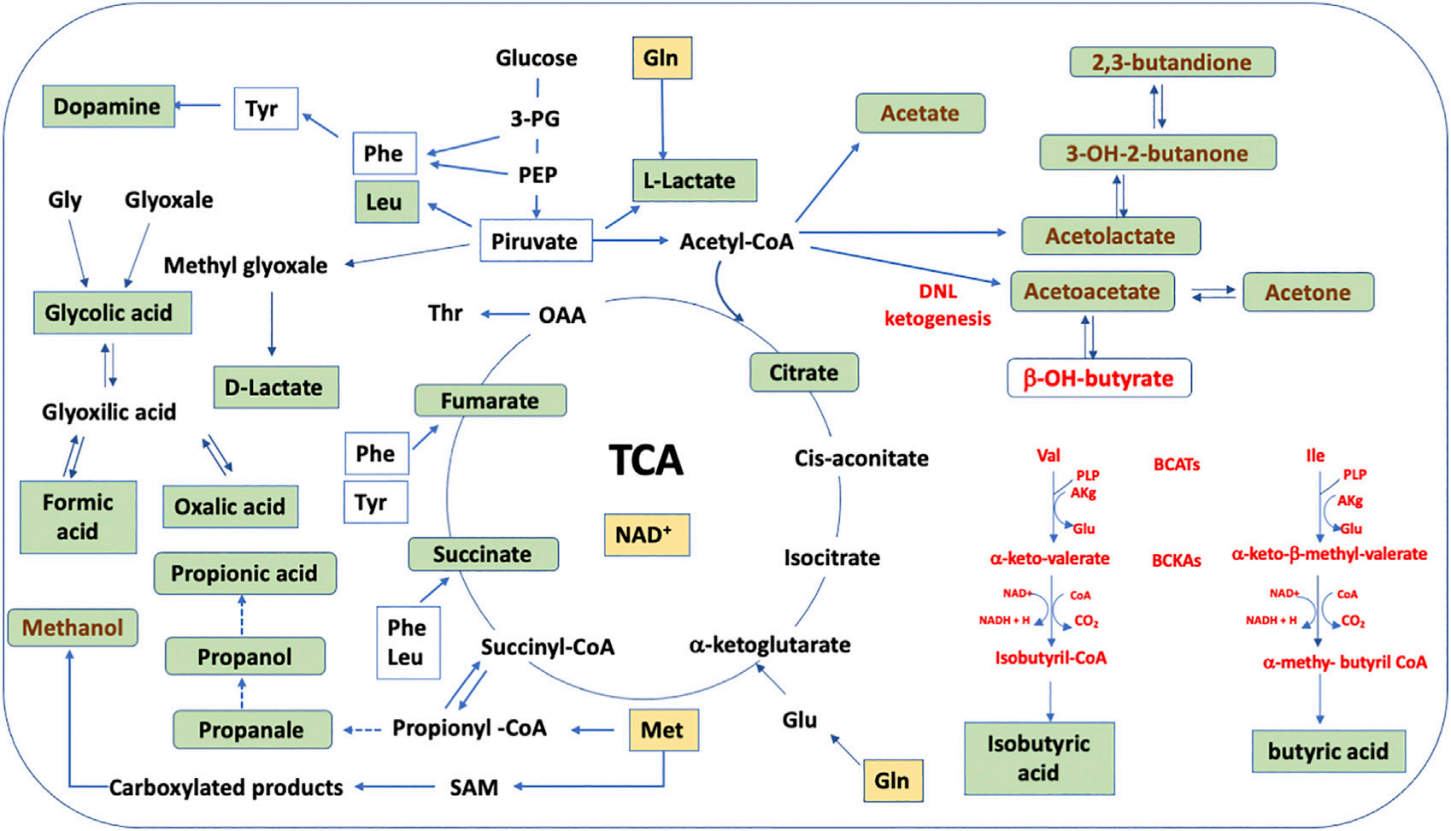
Schematic representation of the HN9.10e cell line metabolism hypothesized on the base of the analysis of CCM. The key metabolites that significantly increase or decrease are shown and are represented inside the neuron. Their finding in the CCM supposes specific or aspecific transport outside the cells. BCATs, branched chain amino acids; BCKAs, branched chain *α*-keto acids; DNL, *de novo* lipogenesis; yellow box, decreased concentration; green box, increased concentration; red pathways, if excess of acetyl-CoA occurs; brown pathways, if deficit of NAD^+^ and acetyl-CoA occurs.

HS-SPME-GC-MS data (Figure 2; Supplementary Table S3) support HPLC-DAD data. Only one volatile organic compound (butanal) significantly decreased after cell culturing, likely due to its reduction to butanol. Correspondingly, after cell culturing, 2-methyl-butanol, 3-methyl-butanol, and 2-methyl-propanol increased for the reduction of the corresponding aldehyde. This result is not surprising considering the potential impact of VOC in plastic culture flasks on cell metabolism (Filipiak et al., 2016; Chu et al., 2020). More interestingly, we observed after neuron culturing a significant increase in other nine VOCs: 3-OH-2-butanone (acetoin), 2,3-butanedione, propanal and 2-methyl-propanal, methanol and methylacetate, propanol, 2-pentanol, and acetone. Potential pathways that involve the metabolites determined by HPLC-DAD and HS-SPME-GC-MS and that significantly increase or decrease their concentration in the CCM are schematized in Figure 4, assuming specific or aspecific transport outside the cells.

While 3-OH-2-butanone (acetoin), 2,3-butanedione, and acetone are products of ketone body metabolism, the increase of propanal and propanol, 2-methylpropanal, methanol and methylacetate, and 2-pentanol is interesting. Propanol can be derived from the reduction of propanal that, in its turn, may be derived from propionyl-CoA. These reactions are typical and well known in bacterial metabolism (Goldberg et al., 2006). However, these compounds, aldehydes and alcohols, are likely formed due to the dehydrogenase activity characteristic of cell proliferation in normal cells during tissue regeneration (Muzio et al., 2012).

We previously found that ethanol is produced in the HN9.10e cell line (Colombaioni et al., 2017b). Ethanol and methanol were found in the plasma of healthy women and men after intake of the specific ADH inhibitor 4-methylpyrazole (4-MP) administered by intraperitoneal injection to bypass the gastrointestinal tract, in order to show that the inhibition was related to liver ADH rather than the ADH of the intestinal microflora (Komarova et al., 2014). Recently, methanol-sensitive genes have been identified (Dorokhov et al., 2015). The increase of methanol and methylacetate (the latter likely derived from the reaction of methanol with acetic acid) in the CCM after 4 days of HN9.10e cell line culturing is suggestive of its endogenous production likely from the hydrolysis of the methyl ester bond of the proteins methylated by S-adenosylmethionine (SAM) activity (Lee et al., 2008). Its increase correlates with the increase of formic acid formed from methanol oxidation into formaldehyde which, in turn, is oxidized into formic acid by alcohol dehydrogenase and aldehyde dehydrogenase, in agreement with literature data (Lee et al., 2008).

## Conclusion

Toxicology aims to protect human health from exposure to toxic chemicals in the environment, and pharmacology aims to investigate the effect of drugs. If compared to studies based on animal models or human subjects, extracellular metabolomics can easily and more ethically provide new insights into the molecular basis of toxic agents or drugs. However, reliable results should be based on a solid knowledge of normal cell metabolism, which represents the starting point to assess the cell response to external stimuli. While extraction methods are generally selective and destructive, the analysis of CCM allows to operate on the same cell culture batch and to limit the perturbation of the cell system. Different batches may indeed compromise the robustness and reproducibility of the results.

The study proposed provides a fast protocol (HPLC-DAD and HS-SPME-GC-MS analysis) able to monitor the extracellular metabolic profile of cell culture lines with minimal perturbation of the cell system. The data obtained on six different flasks evidenced also a satisfactory reproducibility.

This approach, which combines common laboratory equipment and the analysis of CCM, could also help to develop standardized, compliance procedures to generate cultured cell samples for further metabolomic studies. In our operating condition, HN9.10e immortalized neurons (1 ml 330.00 cell/ml seeded in 5 ml of fresh CCM) reached 41 ± 1% confluence in 4 days, and the targeted analysis of the CCM by liquid chromatography with diode array detection and HS-SPME-GC-MS allowed the reproducible identification and quantification of 23 polar metabolites and 19 volatile metabolites, respectively (*N* = 6 independent flasks). Our results suggest that HN9.10e immortalized neurons are a relevant cell culture model and that the selected operating conditions guarantee a stability of the cell metabolic state.

An accurate study of the metabolic profile of the HN9.10e cell line has never been performed before, and it could represent a quality parameter before any other targeting assay or further exploration.

## Data Availability

The original contributions presented in the study are included in the article/Supplementary Material. Further inquiries can be directed to the corresponding author.
