# Supplementary material for: Unraveling the Extracellular Metabolism of Immortalized Hippocampal Neurons Under Normal Growth Conditions
Source: Front Chem. 2021 Apr 16;9:621548. doi: 10.3389/fchem.2021.621548 (PMC8085660; doi:10.3389/fchem.2021.621548)
Supplement: Supplementary file 1 [file datasheet1.pdf]

SUPPLEMENTARY INFORMATION

Unraveling the extracellular metabolism of immortalized hippocampal neurons under normal growth conditions

Beatrice Campanella[1], Laura Colombaioni[2¶], Riccardo Nieri[1], Edoardo Benedetti[3¶], Massimo Onor[1¶], Emilia Bramanti[1¶*]

[1] National Research Council, Institute of Chemistry of Organometallic Compounds (CNR-ICCOM), Pisa, Italy.

[2] National Research Council, Institute of Neuroscience (CNR-IN), Pisa, Italy.

[3] Hematology Unit, Department of Oncology, University of Pisa, Pisa, Italy.

***Corresponding author**

Emilia Bramanti bramanti@pi.iccom.cnr.it

Tel: 0039-050-315-2293. Fax: +39-050-315-2555

ORCID Emilia Bramanti: orcid.org/0000-0001-8478-7370

Table S1. Details of the HS-SPME-GC-MS method used in this work.

Instrumentation	CTC CombiPAL autosampler
	Agilent 6850 gas chromatograph
	Agilent 5975c mass spectrometer
**HS experimental conditions**	
Vial Incubation Temperature	50°C
Vial incubation Time	10 min
SPME Fiber	85 µm carboxen/polydimethylsiloxane (CAR/PDMS)
SPME Exposure Time	10 min
SPME Desorption Temperature	280°C
SPME Desorption Time	15 min 0.1ml/min splitless
	15 min 300 ml/min for SPME fiber cleaning
**GC experimental conditions**	
Analytical column	DB-WAX-UI
	30 m × 0.25 mm, 0.5 um film thickness
Liner	ultra-inert SPME liner (1 mm i.d.)
Injection	Splitless
GC column flow	Helium 1 mL/min
Oven	30 °C for 10 min, 5 °C/min up to 60 °C (2 min hold time), then 10°C/min up to 240°C (5 min hold time)
Run time	41 min
**MS experimental conditions**	
Ionisation Mode	EI
Transfer Line Temperature	240°C
Ion Source Temperature	250°C
Dwell Time	100 ms
Acquisition	Both SIM and fullscan (20-200 M/z)

Table S2. RP-HPLC-DAD method

Instrumentation	Agilent 1260 Infinity HPLC system (G1311B quaternary pump) equipped with 1260 Infinity High Performance Degasser,
	Agilent 1260ALS autosampler (G1329B)
	Agilent UV/vis diode array (1260 DAD G4212B)
Column	Zorbax Eclipse Plus Phenyl-Hexyl RP Analytical 959990-912 (4.6 x 250 mm) (Agilent Tech.) (silica particle size 5 µm)
Mobile phase	(A) 5 mM sulphuric acid, pH 2.2
	(B) methanol
Column Temperature	45°C
Elution	15 min 100% A, linear gradient up to 20% A - 80% B in 10 min, 10 min 20% A - 80% B
Flow rate	0.8 mL/min
Injection volume	5 µL
Detection	220 nm

Table S3. List of the analytes quantified by HS-SPME-GC-MS with the relative retention time, quantifier and qualifier mass-to-charge ratio, and average concentration in CFCM and CCM.

Analyte	Rt (min)	SIM m/z Quant	SIM m/z Qual	CFCM		CCM	
				Mean (µM)	s.d.	Mean (µM)	s.d.
Acetoin	23.48	45	88	0.030	0.002	0.131	0.016
Acetone	3.92	58	43	0.081	0.014	1.715	0.855
2-methylbutanal	6.90	57	41	0.018	0.001	0.013	0.005
3-methylbutanal	7.14	44	58	0.016	0.001	0.016	0.007
Butanedione	11.14	43	86	0.004	0.001	0.020	0.002
Butanale	5.52	72	44	0.117	0.001	0.046	0.023
Butanol	19.86	56	31	0.195	0.008	0.261	0.016
2-methylbutanol	21.70	57	41	0.005	0.000	0.047	0.003
3-methylbutanol	21.73	55	42	0.008	0.000	0.098	0.007
2-Butanone	6.41	72	43	0.066	0.006	0.313	0.212
Hexanal	16.39	56	44	0.010	0.000	0.017	0.009
Methanol	6.57	31	32	5.323	0.381	9.085	0.699
Methylacetate	4.23	74	43	0.001	0.000	0.002	0.000
2-pentanol	18.92	45	55	0.026	0.000	0.092	0.026
Propanal	3.45	58	29	0.021	0.001	0.114	0.005
2-methylpropanal	3.83	72	41	0.017	0.001	0.074	0.024
Propanol	14.85	31	59	0.078	0.002	3.044	0.561
2-methylpropanol	17.59	43	41	0.039	0.003	4.906	0.770
2-butanol	14.22	45	59	0.026	0.001	0.038	0.029
Ethanol $^{13}C_2$ (ISTD)	8.70	47	32				
Acetone $^{13}C_2$ (ISTD)	3.92	60	44				

Table S4. Retention times, fitting parameters (slope and standard deviation SD of the slope), correlation coefficients of the calibration plots and limit of detection (LOD) of 25 metabolites potentially quantifiable by RP-HPLC-DAD inCCM (V_{inj}=5 μL; N=3 replicates). Recovery was calculated with respect to the slope of the calibration curve obtained in 5 mM suphuric acid. SD= standard deviation.

Compound name	HMDB ID	Retention time (min)	Slope in 5 mM sulfuric acid (peak area mM^{-1})	Slope in CCM (peak area mM^{-1})	Recovery %	LOD in CCM (μM)
Acetic acid	HMDB0000042	5.095	46 ± 0.1	45.9 ± 6.6	99.7	3.30
Acetoacetic acid	HMDB0000060	7.114	70 ± 7	78.4 ± 1.8	112.1	1.70
Butyric acid	HMDB0000039	23.187	66 ± 0.2	70 ± 2.0	106.1	1.91
Citrate	HMDB0000094	5.66	345 ± 1.5	355.3 ± 45.8	103.0	0.42
Formic acid	HMDB0000142	3.723	63 ± 0.1	64.8 ± 11.1	102.9	2.42
Fumaric acid	HMDB0000134	6.184	20940 ± 29	19700.0 ± 3341.4	94.1	0.01
Glycolic acid	HMDB0000115	3.564	58 ± 1.5	54.1 ± 4.3	93.3	2.61
Isobutyric acid	HMDB0001873	22.94	102 ± 2.0	101.2 ± 7.0	99.2	1.38
L-Cysteine	HMDB0000574	3.608	183 ± 1.3	180.4 ± 29	98.6	0.86
Dopamine	HMDB0000073	5.383	11333 ± 45	10500.0 ± 1300.0	92.6	0.01
L-Glutamine	HMDB0000641	3.068	184 ± 1.0	176.0 ± 14.0	95.7	0.80
L-Glutamic acid	HMDB0000148	3.141	105 ± 1.0	120.2 ± 22.3	114.5	1.33
L-Histidine	HMDB0000177	2.855	7209 ± 84	6836.7 ±578.8	94.8	0.02
Lactic acid	HMDB0000190	4.693	110 ± 0.3	110.8 ± 15.3	100.8	1.36
L-Leucine	HMDB0000687	8.635	67 ± 0.3	73.8 ± 10.1	110.1	2.04
L-Methionine	HMDB0000696	5.855	1278 ± 2.0	1186.7 ± 251.7	92.9	0.14
L-Phenylalanine	HMDB0000159	18.104	3328 ± 5.0	3196.7 ± 134.8	96.1	0.040
L-Threonine	HMDB0000167	3.121	57 ± 1.0	58.8 ± 8.4	103.1	2.58
L-Tryptophan	HMDB0000929	23.572	63966 ± 362	64893.3 ± 1958.1	101.4	0.003
L-Tyrosine	HMDB0000158	7.718	16717 ± 25	16678.3± 327.0	99.8	0.008
NAD^+	HMDB0000902	4.109	19020 ± 30	17440.0 ± 940.6	98.9	0.01
Oxalic acid	HMDB0002329	3.265	1223 ± 22	1245.3 ± 185.4	101.8	0.12
Propionic acid	HMDB0000237	10.372	51 ± 0.1	46.7 ± 6.9	91.6	3.26
Pyruvic acid	HMDB0000243	4.392	959 ± 1.6	972.9 ± 126.9	101.4	0.15
Succinic acid	HMDB0000254	6.886	88 ± 0.9	86.7 ± 12.9	98.5	1.76

Table S5. RP-HPLC-DAD chemical characterization of cell free DMEM-F12 Dulbecco culture media (D6421) (N=6 replicates). The concentration of several metabolites found in operating conditions is compared with the composition declared by the producer. NAD^+= oxidized nicotinamide adenine dinucleotide.

Compound	t_R *(min)*	*Declared concentration (μM)*	*Concentration_found mean ± SD (μM)*	*CV%*	*Recovery %*
L-Histidine	2.880	150.2	175.4 ± 30.1	17.2	117.0
L-Glutamine	3.216	4000	3843.2 ± 616.6	96.1	100.9
L-Cysteine	3.536	100	106.8 ± 31.3	29.1	106.8
NAD^+	4.109	16.5	11.1 ± 3.1	28.1	67.1
Pyruvate	4.340	500.0	411.1 ± 95.7	23.3	82.2
L-Methionine	5.855	115.5	110.6 ± 32.6	29.5	95.7
L-Tyrosine	7.718	212.0	189.6 ± 48.3	25.5	89.5
L-Leucine	8.635	450.2	266.6 ± 77.0	29.0	59.1
L-Phenylalanine	18.104	214.8	208.7 ± 47.7	22.9	97.2
L-Tryptophane	23.525	44.2	42.2 ±7.9	18.6	95.5

Table S6. CCM composition determined by RP-HPLC-DAD after 4 day HN9.10e cell line culturing (N=6 replicates).

Metabolite	t_R	*Minimum (μM)*	*Maximum (μM)*	*Mean ± SD (μM)*	*IQR*	*CV%*	*Concentration in cell free CCM (μM)*
L-Histidine	2.880	81.8	98.8	94.8± 6.5	3.2	6.9	175.4 ± 30.1
L-Threonine/<br>L-Glutamic acid*	3.120*	nd	nd	nd	nd	nd	450**<br>50**
L-Glutamine	3.216*	nd	nd	nd	nd	nd	3843.2 ± 613.6
Oxalic acid	3.265	58.8	108.6	85.3± 20.0	28.0	23.4	< LOD
L-Cysteine	3.458	101.1	278.7	162.6± 74.9	95.6	46.1	106.8 ± 31.1
Glycolic acid	3.608	2577.6	3060.3	2721.3± 171.4	43.1	6.3	< LOD
Formic acid	3.78	261.9	428.6	337.3± 60.0	67.5	17.8	< LOD
NAD^+	4.109	2.1	2.9	2.6± 0.3	0.3	11.6	11.1 ± 3.1
Pyruvate	4.34	531.8	677.8	632.1± 53.4	40.4	8.5	411.1 ± 95.7
Lactic acid	4.693	14759.1	18350.0	17138.6± 1257.4	728.4	7.3	< LOD
Acetic acid	5.095	5260.9	7010.9	6054.3± 820.3	1383.2	13.5	< LOD
Dopamine	5.383	2.4	4.8	3.8± 1.0	1.4	25.2	< LOD
Citrate	5.66	129.0	171.0	161.6± 16.6	8.7	10.3	< LOD
L-Methionine	5.932	< LOD	< LOD	< LOD	< LOD	< LOD	110.6 ± 32.6
Fumaric acid	6.184	15.9	19.2	17.5± 1.5	2.4	8.4	< LOD
Succinic acid	6.886	267.0	534.1	350.4± 111.4	129.3	31.8	< LOD
Acetoacetic acid	7.122	8485.7	10307.1	9786.9± 655.8	200.0	6.7	< LOD
L-Tyrosine	7.718	108.8	132.0	124.1± 8.0	2.4	6.4	189.6 ± 48.3
L-Leucine	8.635	664.2	798.5	711.4± 45.7	14.9	6.4	266.0 ± 77.7
Propionic acid	10.372	833.3	1117.6	936.3±126.6	171.6	13.5	< LOD
L-Phenylalanine	18.104	181.9	228.5	214.8± 17.0	9.9	7.9	208.7 ± 47.7
L-Isobutyric acid	22.94	745.1	1029.4	908.5± 92.2	29.4	10.1	< LOD
L-Butyric acid	23.187	378.8	833.3	599.7± 157.6	153.4	26.3	< LOD
L-Tryptophane	23.525	30.8	37.7	35.2± 2.3	1.0	6.7	42.2± 7.9

* Not determined due to close retention times. ** declared

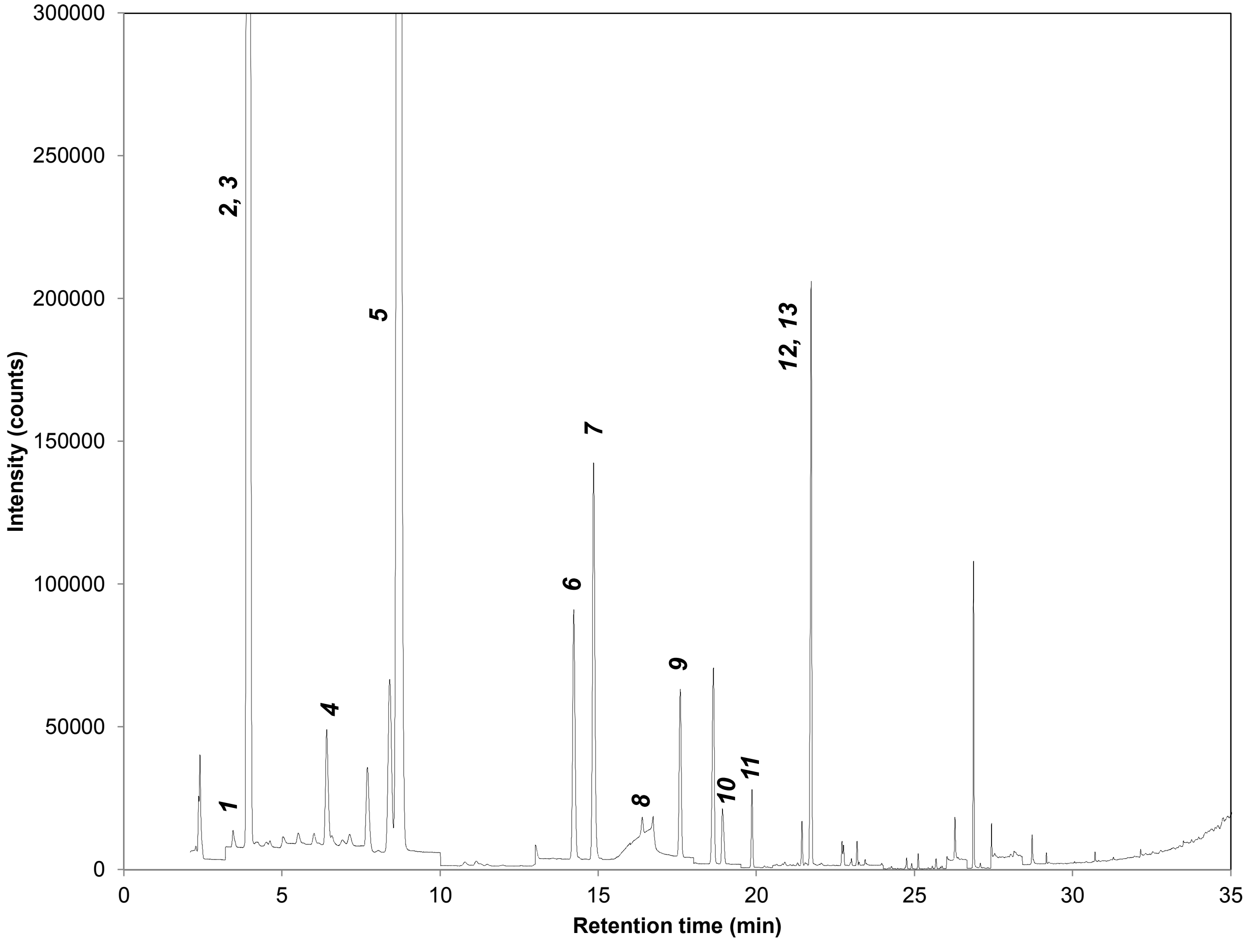

Figure S1. Representative HS-GC-MS totali on chromatograms of a CCM sample acquired in SIM full scan mode (1 - propanal (3.4 min); 2, 3 - acetone, acetone $^{13}C_2$ (3.9 min); 4 – 2-butanone (6.4 min); 5 – ethanol $^{13}C_2$ (8.7 min); 6 – 2-butanol (14.2 min); 7 – propanol (14.8 min); 8 – hexanal (16.4 min); 9 – 2-methylpropanol (17.5 min); 10 – 2-pentanol (18.9 min); 11 – butanol (19.8 min); 12, 13 – 2-methylbutanol, 3-methylbutanol (21.7 min).

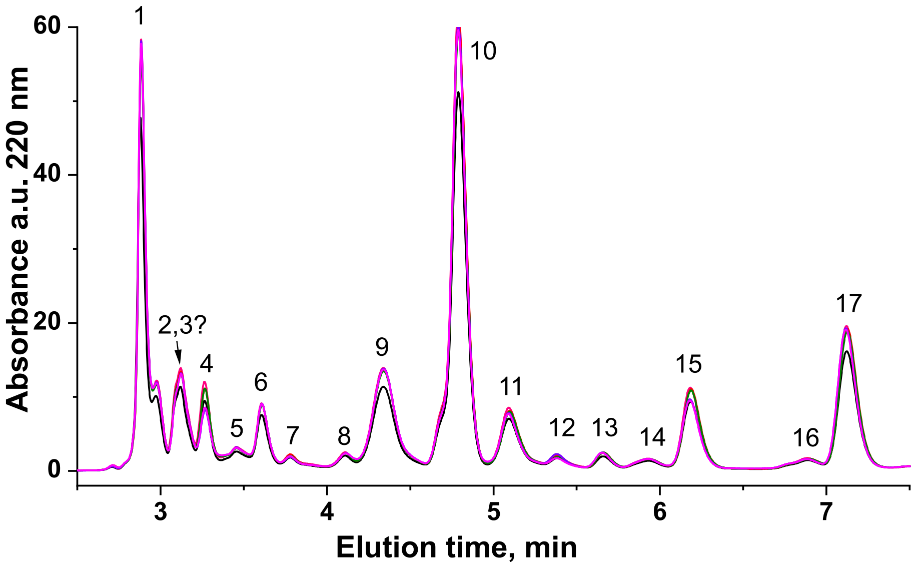

(A)

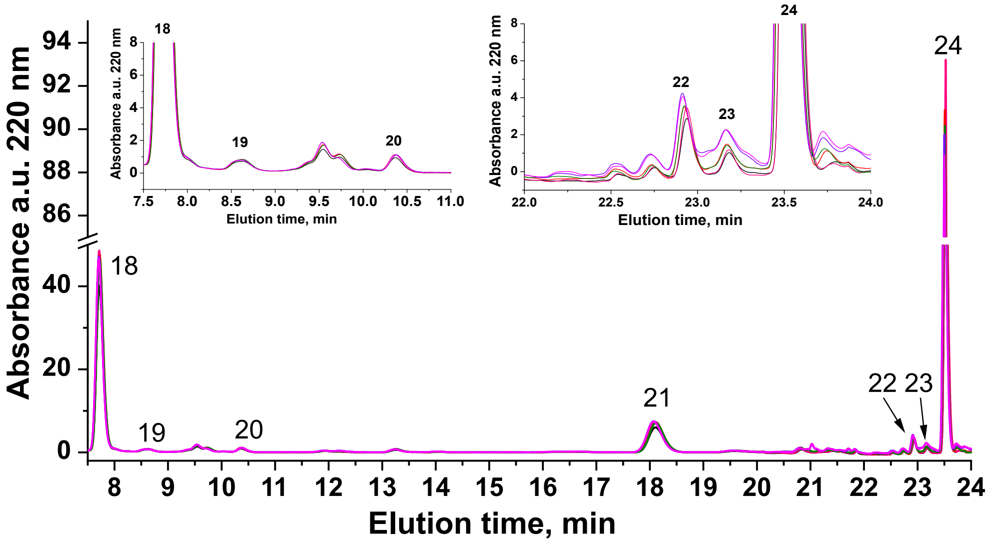

(B)

Figure S2. Absorbance chromatogram at 220 nm of CCM (V_{inj} =5 μL) after 4 days HN9.10e cell line culturing (N=6 replicates in different flasks). The chromatogram of the blank has been subtracted. 1= L-Histidine; 2= L-Serine; 3= L-Threonine/L-Glutamic acid*; 3= L-Glutamine; 4= Oxalic acid; 5= L-Cysteine; 6= Glycolic acid; 7= Formic acid; 8= NAD^+; 9= Pyruvate; 10= Lactic acid; 11= Acetic acid; 12= Dopamine; 13= Citrate; 14= L-Methionine; 15= Fumaric acid; 16= Succinic acid; 17= Acetoacetic acid; 18= L-Tyrosine; 19= L-Leucine; 20= Propionic acid; 21= L-Phenylalanine; 22= Isobutyric acid; 23= Butyric acid; 24= L-Tryptophane.
